# In this month’s Bulletin

**DOI:** 10.2471/BLT.23.000223

**Published:** 2023-02-01

This month’s theme is linked to the Prince Mahidol Award Conference on the intersection of health with climate change, environment and biodiversity. 

In the editorial section, Viroj Tangcharoensathien et al. (82) discuss the political commitments needed to mitigate the health impact of the climate crisis. Sarah Whitmee et al. (83) introduce a new database that tracks global carbon emissions. 

Fid Thompson (86–87) reports on urban planning changes needed to address climate change, flooding and sanitation. Joy Shumake-Guillemot tells Gary Humphreys (88–89) about recent climate and health initiatives in the World Meterological Organization.

## Algeria, Bangladesh, Benin, Brazil, Burkina Faso, Congo, Côte d'Ivoire, Egypt, Gabon, Ghana, Guatemala, Guinea, Honduras, India, Kenya, Mali, Mauritius Morocco, Namibia, Senegal, Togo, Tunisia, Uganda, United Republic of Tanzania, Zambia 

### Preventing hunger and thirst 

Sera L Young et al. (90–101) investigate associations between food and water insecurities.

## Cambodia

### Wild food environments and food security 

Swetha Manohar et al. (140–148) describe how healthy livelihoods depend on the Mekong River.

## Eastern Mediterranean Region, Algeria

### Managing risks of SARS-CoV-2 transmission and severe disease

Zlatko Nikoloski et al. (111–120) measure adherence to public health and social measures. 

## India

### Coastline, forests and energy 

Adithya Pradyumna et al. (149–151) examine mitigation measures in terms of climate justice.

### Counting deaths and establishing cause 

Nandita Saikia et al. (102–110) study factors associated with registration coverage.

## Western Pacific Region

### Health and economic benefits of clean air 

Nicole Egerstrom et al. (130–139) estimate lives and resources saved if guidelines were met.

## Global

### Improving urban agricultural biodiversity

Mary C Sheehan (121–129) analyses climate adaptation plans of large cities.

### More frequent tropical cyclones 

Andrea Monica D Ortiz et al. (152–154) list requirements for cyclone recovery.

### Realizing health and climate co-benefits

Laurie Laybourn-Langton (155–157) calls for justice in climate negotiations. 

### Joining the UN Framework Convention on Climate Change 

Jen Iris Allan (158–160) describes the trade-offs in linking health to climate change advocacy.

